# Mumps Outbreak in Shivamogga, India (2023–2024): Age-Specific Attack Rates, Complications, and an Economic Case for Measles-Mumps-Rubella Vaccine Inclusion in the Universal Immunization Programme

**DOI:** 10.7759/cureus.101201

**Published:** 2026-01-09

**Authors:** Srujana Gireesh, Vikram Sakaleshpur Kumar, Ramya S R

**Affiliations:** 1 Paediatrics and Child Health, Subbaiah Institute of Medical Sciences, Shivamogga, IND; 2 Microbiology, Subbaiah Institute of Medical Sciences, Shivamogga, IND

**Keywords:** attack rate, cost, india, mmr, mumps, outbreak, pediatrics, policy, vaccination

## Abstract

Background

Recurrent mumps outbreaks continue to be reported in India, where the vaccine is not part of the national immunization schedule. Robust, age‑specific burden data from outbreak settings remain limited.

Methods

We conducted a retrospective investigation of pediatric mumps during epidemiologic weeks (EW)49‑2023 to EW8‑2024 across Subbaiah Institute facilities in Shivamogga, Karnataka, India. Clinically suspected cases were line-listed; laboratory confirmation used anti-mumps IgM enzyme-linked immunosorbent assay (ELISA). We described demographics, complications, and length of stay (LOS) among IgM‑confirmed cases, estimated age‑specific attack rates using district population denominators, and compared complication risks by age with exact tests and risk ratios (RRs; 95% CI). Vaccination status was ascertained from immunization cards/registers where available, otherwise by parental recall.

Results

Among approximately 2,500 clinically suspected cases during EW49-2023 to EW8-2024, 318 children (median age 6.5 years (IQR five to nine years); 49% female) were IgM-confirmed. The overall attack rate among children aged one to 16 years was 69 per 100,000 (95% CI 61.6-77.0), highest in the five-to-nine-year-old group (147 per 100,000) compared with <5 years (70 per 100,000) and ≥10 years (13 per 100,000). Nine children (2.8%, 95% CI 1.3-5.2) developed complications such as pancreatitis (0.9%), aseptic meningitis (0.9%), orchitis (0.6%), and oophoritis (0.3%). Complication risk increased with age: 0% in <5 years, 1.9% in five to nine years, and 19.2% in ≥10 years (RR 10.1, 95% CI 3.0-34.3; Fisher’s p < 0.001). All confirmed cases were unvaccinated for mumps. Median hospital stay was six days (IQR three to seven days) for complicated vs. three days (IQR two to four days) for uncomplicated cases.

Conclusion

This outbreak disproportionately affected school‑aged children, with complications concentrated in those ≥10 years. Findings quantify the preventable disease burden and support inclusion of a two-dose measles-mumps-rubella (MMR) vaccination schedule in India’s Universal Immunization Programme (UIP), along with a catch-up vaccination strategy for children and adolescents aged between five and 15 years. Observed hospitalization burden highlights potential economic relevance; however, no formal cost-effectiveness or budget-impact analysis was performed.

## Introduction

Mumps is a contagious viral illness caused by a single-stranded RNA virus of the Paramyxoviridae family, typically presenting with an abrupt onset of tender swelling of the parotid or other salivary glands [1]. Although only one serotype exists, molecular studies have identified at least 13 genotypes based on the SH gene sequence variation [2]. Live-attenuated mumps-containing vaccines using strains such as Jeryl-Lynn, RIT 4385, Leningrad-3, Leningrad-Zagreb, Urabe Am9, S79, and Rubini have been available since the 1960s [3,4]. In India, the Leningrad-Zagreb strain predominates in domestic MMR formulations [4,5].

Transmission occurs via respiratory droplets or saliva, with infectivity roughly from two days before to five days after onset of parotid swelling [5,6]. Mumps can lead to several complications, including orchitis, oophoritis, mastitis, pancreatitis, meningitis, encephalitis, sensorineural hearing loss, nephritis, myocarditis, paralysis, seizures, and cranial nerve palsies [2,6]. Despite low case fatality, these complications impose substantial morbidity and economic costs, particularly when outbreaks disrupt schools and health services [2].

Globally, mumps has resurged in the last decade, especially where two-dose measles-mumps-rubella (MMR) coverage is suboptimal, or immunity has waned in adolescent and young adult cohorts [2,4]. In India, the Universal Immunization Programme (UIP) currently excludes the mumps vaccine, although measles-rubella (MR) is offered, and mumps-containing vaccines remain largely confined to the private sector. Uptake, therefore, varies by household income, healthcare-seeking behavior, and proximity to urban private facilities. Children from lower socio-economic backgrounds, who predominantly depend on public health services, are consequently less likely to receive MMR than children from higher socio-economic groups accessing private pediatric care. Under-notification of mumps cases, particularly milder or uncomplicated infections that do not seek care or are managed outside reporting facilities, is likely to result in underestimation of true incidence. At the same time, preferential capture of more severe or complicated cases in hospital-based surveillance may inflate the apparent proportion of complications among reported cases, thereby biasing complication estimates upward. Routine surveillance is weak, as mumps is not nationally notifiable; yet recurrent outbreaks are reported through the Integrated Disease Surveillance Programme (IDSP) [7-9]. Evidence quantifying age-specific attack rates, complication risk, and laboratory-verified non-vaccination in Indian outbreak settings remains sparse.

We investigated a pediatric mumps outbreak in Shivamogga District, Karnataka, India, during 2023-2024. Our objectives were to (i) describe the epidemic curve and clinical profile of IgM-confirmed cases; (ii) estimate age-specific attack rates using district population denominators; (iii) compare complication risks across age bands using exact statistics; and (iv) provide a first-pass budget-impact rationale for adding two-dose MMR with school-age catch-up to India’s UIP.

## Materials and methods

Study design and setting

We conducted a retrospective outbreak investigation at the Department of Pediatrics, Subbaiah Institute of Medical Sciences and Research Centre, Shivamogga District, Karnataka, India. The study period encompassed epidemiologic weeks (EW) 49-2023 to 8-2024 (December 2023-February 2024), during which a district-wide surge in clinically suspected pediatric mumps was reported across outpatient (OPD) and inpatient (IPD) services. The hospital serves as a major pediatric referral center for Shivamogga District and neighboring blocks.

**Figure 1:**
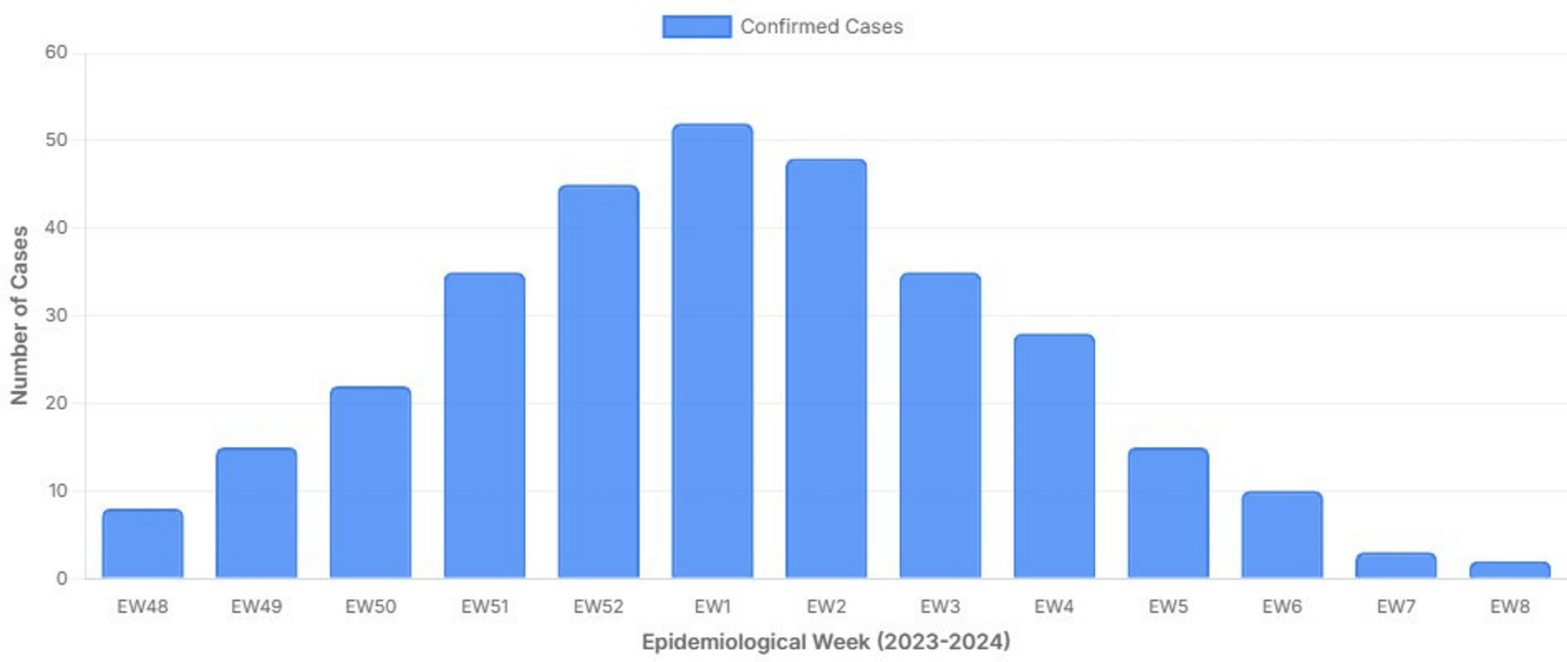
Epidemic curve of IgM-confirmed mumps cases by epidemiologic week (EW) in Shivamogga district between 2023–2024 with a seven-day moving average. The x-axis displays EW numbers from EW49–EW52 (2023), followed by EW1–EW8 (2024); EW1 follows EW52 because EWs are numbered sequentially across calendar years. The y-axis shows the number of IgM-confirmed cases. (Source: WHO, UNICEF, ACVIP data visualized by authors, Subbaiah Institute case data; licensed under CC-BY 4.0.) [10,11,12]. WHO: World Health Organization; UNICEF: United Nations Children's Fund; ACVIP: Advisory Committee on Vaccines and Immunization Practices

The epidemic curve for IgM-confirmed cases by EW is shown in Figure 1, which illustrates the transition from late 2023 (EW49-EW52) into early 2024 (EW1-EW8), with EW1 following EW52 as per the standard EW convention.

Case definition and ascertainment

Case definition followed the World Health Organization (WHO) criteria for mumps: “acute onset of unilateral or bilateral, tender, self-limited swelling of the parotid or other salivary glands, lasting two or more days without other apparent cause” [13].

Subbaiah Institute of Medical Sciences functions as a major pediatric referral center for Shivamogga district and receives suspected mumps cases from surrounding taluks through both government and private referral pathways. However, it is not the sole facility managing suspected mumps cases in the district, and not all government or IDSP-linked facilities routinely refer cases or laboratory samples to our center. Consequently, case ascertainment in this study represents facility-based surveillance rather than complete district-wide capture.

Serum samples were collected between days 3 and 10 after onset of parotitis and tested for anti-mumps IgM by enzyme-linked immunosorbent assay (ELISA) using the EUROIMMUN Anti-Mumps-Virus ELISA (IgM) (EUROIMMUN Medizinische Labordiagnostika AG; Lübeck, Germany; Catalogue no. EI 2630-9601 M; Lot no. 20231015A; expiry 09/2024). Assays were performed according to the manufacturer’s instructions. Samples with an optical density index (OD index) ≥1.1 were considered positive per the kit insert. The kit’s reported sensitivity and specificity are 93% and 98%, respectively (manufacturer instructions for use). Quality control included kit-provided positive and negative controls and internal laboratory controls. The IgM-negative results do not fully exclude mumps infection, particularly when samples are collected early or in previously exposed individuals.

Children with parotid swelling due to non-viral or non-infectious causes, such as bacterial parotitis, ductal obstruction, or autoimmune diseases, were excluded. A total of 318 laboratory-confirmed cases fulfilling both clinical and serological criteria were included in the final analysis.

During the outbreak, laboratory confirmation with anti-mumps IgM ELISA was performed on a subset of clinically suspected cases, prioritizing hospitalized children and those with complications, atypical presentations, or referral cases, in line with routine outbreak investigation practice and laboratory capacity constraints. Mild, self-limiting cases managed on an outpatient basis were not uniformly tested.

Data collection

For each confirmed case, demographic and clinical data were retrieved from OPD, IPD, and laboratory records. Variables included age, sex, residence, date of symptom onset, presence and laterality of parotitis, complications, vaccination status, and length of stay (LOS; for admitted cases).

Complications were defined as follows: Pancreatitis: elevated serum amylase or lipase with compatible abdominal symptoms; Aseptic meningitis: cerebrospinal fluid pleocytosis with negative bacterial culture and compatible clinical presentation; Orchitis or oophoritis: clinical findings corroborated by ultrasonography; Other systemic involvement considered plausibly mumps-related by treating clinicians.

Population denominators and attack rates

Age-specific population denominators for Shivamogga District were derived from the 2011 Census of India [13] and projected to 2023 using state-level demographic growth rates. The projected 2023 child population comprised 115,682 children aged <5 years, 143,726 aged five to nine years, and 201,567 aged 10 to 16 years, yielding a total denominator of 460,975 children aged less than 16 years.

Since the case ascertainment was facility-based and restricted to children presenting to Subbaiah Institute facilities, the numerator does not represent complete case capture for the district. Consequently, attack rates calculated using district-wide population denominators should be interpreted as minimum facility-based incidence rates, rather than true population attack rates. These estimates do not adjust for healthcare-seeking behavior, the hospital catchment population, or under-ascertainment of mild or community-managed cases.

Age-specific attack rates were calculated as the number of IgM-confirmed cases per 100,000 population in that age group, with exact (Clopper-Pearson) 95% confidence intervals (CIs). The general formula for the attack rate in an age group is as follows:

Age-Specific Attack Rate

$\text{Estimated Minimum Attack Rate}_i = \frac{C_i}{N_i} \times 100{,}000$

where Ci​ is the number of confirmed mumps cases in age group i; Ni​ is the population denominator for age group i

Overall Pediatric Minimum Attack Rate (0-16 years)

$\text{Attack Rate}_{\mathrm{total}} = \frac{\sum C_i}{\sum N_i} \times 100{,}000$

Statistical analysis

Data were entered into Microsoft Excel (Microsoft Corporation, Redmond, WA, USA) and analyzed using IBM SPSS Statistics version 26 (IBM Corp., Armonk, NY, USA) and R version 4.3 (R Foundation for Statistical Computing, Vienna, Austria). Continuous variables were summarized as medians with IQRs, and categorical variables as frequencies and percentages (N (%)). Differences in complication rates across age groups (<5, five to nine, and ≥10 years) were assessed using Fisher’s exact test. Effect sizes were expressed as RRs with exact 95% CIs. A two-sided p-value < 0.05 was considered statistically significant.

Sensitivity analyses were performed to assess the impact of potential misclassification of vaccination status, including restriction to hospitalized cases, exclusion of recall-only vaccination data, and simulations assuming up to 20% non-differential misclassification toward vaccinated children.

## Results

Outbreak overview and epidemic curve

During EW49-2023 to EW8-2024, approximately 2,500 children presented with clinical features consistent with mumps. Of these, 318/2,500 (12.7%) were confirmed by anti-mumps IgM ELISA and formed the analytic cohort. The epidemic curve by EW, including a seven-day moving average, is shown in Figure 1.

Baseline characteristics are summarized in Table 1. Median age of confirmed cases was 6.5 years (IQR 5-9), and 156/318 (49.1%) were female, while 162/318 (50.9%) were male (Table 1). Parotitis, unilateral or bilateral, was documented in all 318/318 (100.0%) cases. None of the confirmed cases had clinical evidence of alternative diagnoses such as bacterial parotitis.

**Table 1:** Baseline characteristics of IgM-confirmed mumps cases (n = 318) IQR: Interquartile range

Characteristic	Value
Total cases, n (%)	318 (100.0%)
Median age (IQR), years	6.5 (5–9)
Sex, female, n (%)	156 (49.1%)
Sex, male, n (%)	162 (50.9%)
Parotitis present, n (%)	318 (100.0%)
Unvaccinated for mumps, n (%)	318 (100.0%)

Vaccination status for mumps-containing vaccines (e.g., MMR) was verified from immunization cards or health registers when available, and from parental recall when documentation was lacking. No confirmed case had documented evidence of receipt of a mumps-containing vaccine, and parental recall consistently indicated absence of mumps vaccination. We therefore describe vaccination status as undocumented/unreported rather than definitively absent. The source of vaccination data (documented vs recall) was recorded to enable sensitivity analyses (Table 2).

**Table 2:** Vaccination ascertainment source among IgM-confirmed mumps cases (n = 318)

Source of vaccination history	Cases, n	Cases, %	Notes
Verified by card/register	3	0.9%	Card-verified vaccination extremely rare
Parental recall only	315	99.1%	Majority relied on parental recall
Total	318	100.0%	All children unvaccinated for mumps by both sources

Age-specific attack rates

Minimum facility-based attack rates and denominators are presented in Table 3. In the table legend, “population denominator” refers to the projected 2023 child population in each age band in Shivamogga district derived from 2011 Census data [13] and state-level growth rates. Using the projected district child population denominators, the estimated minimum attack rate among children aged less than 16 years was 69 per 100,000 (95% CI 61.6-77.0). The highest burden was observed among children aged five to nine years, with an attack rate of 147 per 100,000 (95% CI 127.7-168.0). In comparison, attack rates were 70 per 100,000 (95% CI 55.6-87.0) in children <5 years and 13 per 100,000 (95% CI 8.4-18.9) in those aged ≥10 years (Table 3).

**Table 3:** Minimum age-specific attack rates per 100,000 children (with 95% confidence intervals) *Population denominator refers to the projected 2023 child population in each age band in Shivamogga district, Karnataka, derived from the 2011 Census of India [13] using state-level demographic growth rates. CI: confidence interval

Age group (years)	Cases, n	Population denominator*	Attack rate per 100,000 (95% CI)
<5	81	115,682	70 (55.6–87.0)
5–9	211	143,726	147 (127.7–168.0)
≥10	26	201,567	13 (8.4–18.9)
Total (1–16)	318	460,975	69 (61.6–77.0)

**Figure 2:**
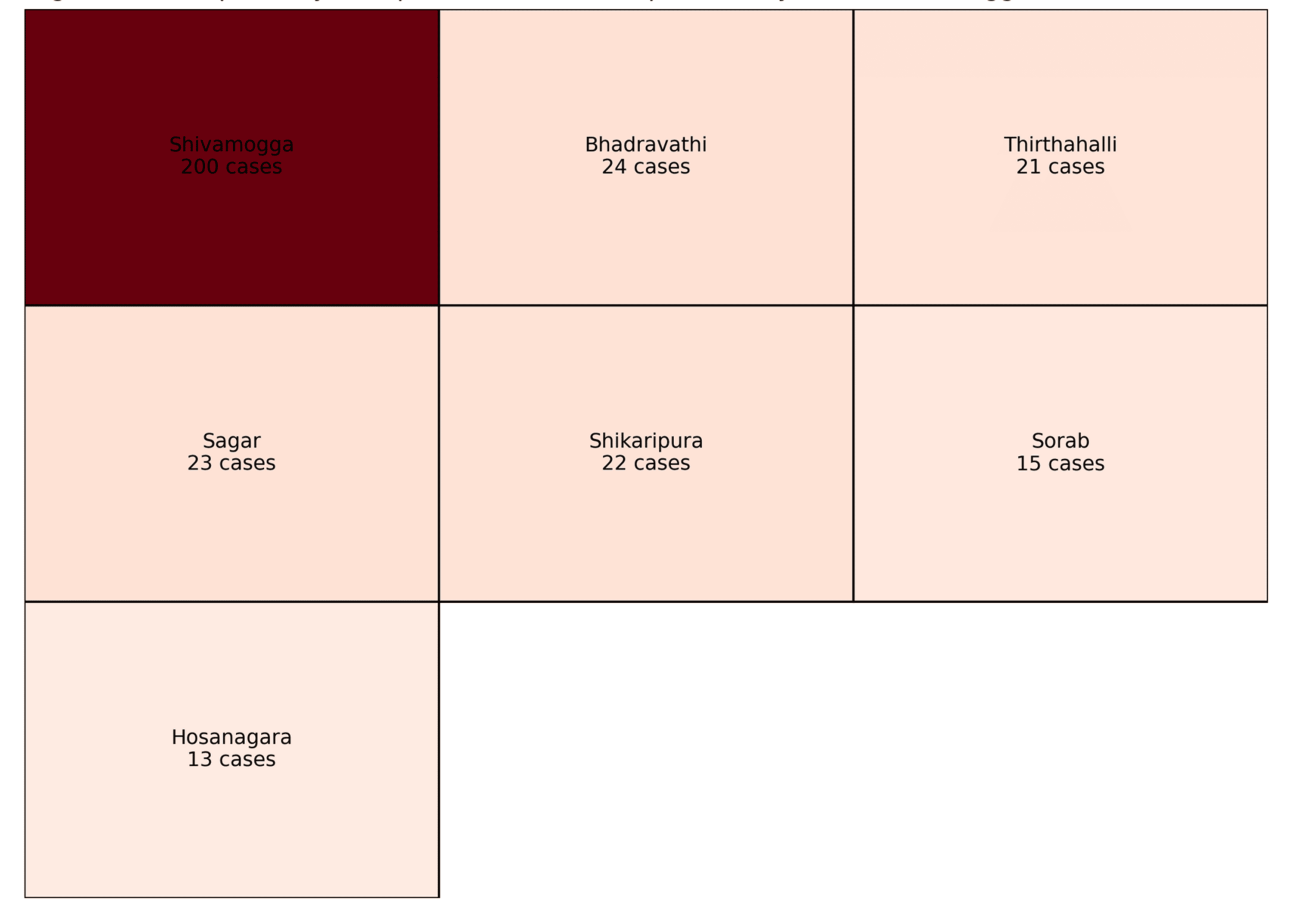
Choropleth style map of confirmed mumps cases by taluk, Shivamogga district in this study(2023-24) Each taluk is shaded according to the number of IgM-confirmed cases, with darker colours indicating higher counts. All mapped data derive from the present outbreak’s line list. Previous Indian mumps outbreaks reported from other states have been described in Integrated Disease Surveillance Programme (IDSP) reports and related studies [8,9].

Spatial distribution of cases by taluk within Shivamogga District is illustrated in Figure 2, a choropleth map in which darker shades represent higher numbers of confirmed cases. All case counts plotted in Figure 2 are derived from the present outbreak line list; prior Indian mumps outbreaks from other states have been described in IDSP reports and related publications [8,9].

Complications and hospital stay

The analytic cohort represents laboratory-confirmed cases among clinically suspected children who underwent testing, rather than all suspected infections. Children with complications had a longer hospital LOS compared with uncomplicated cases: median six days (IQR five to seven days) vs three days (IQR two to four days), respectively (Table 4).

**Table 4:** Complication profile and length of stay by age band LOS: length of stay; IQR: interquartile range

Age group (years)	Total cases, n (%)	Complications, n (%)	Median LOS (days), IQR
<5	81 (25.5%)	0 (0.0%)	3 (2–4)
5–9	211 (66.3%)	4 (1.9%)	3 (2–4)
≥10	26 (8.2%)	5 (19.2%)	6 (5–7)
Total	318 (100.0%)	9 (2.8%)	3 (2–5)

Overall, nine of 318 children (2.8%; 95% CI 1.3-5.2) developed complications (Table 4). These included pancreatitis in three children (0.9%), aseptic meningitis in three children (0.9%), orchitis in two male adolescents (0.6%), and oophoritis in one female adolescent (0.3%).

**Figure 3:**
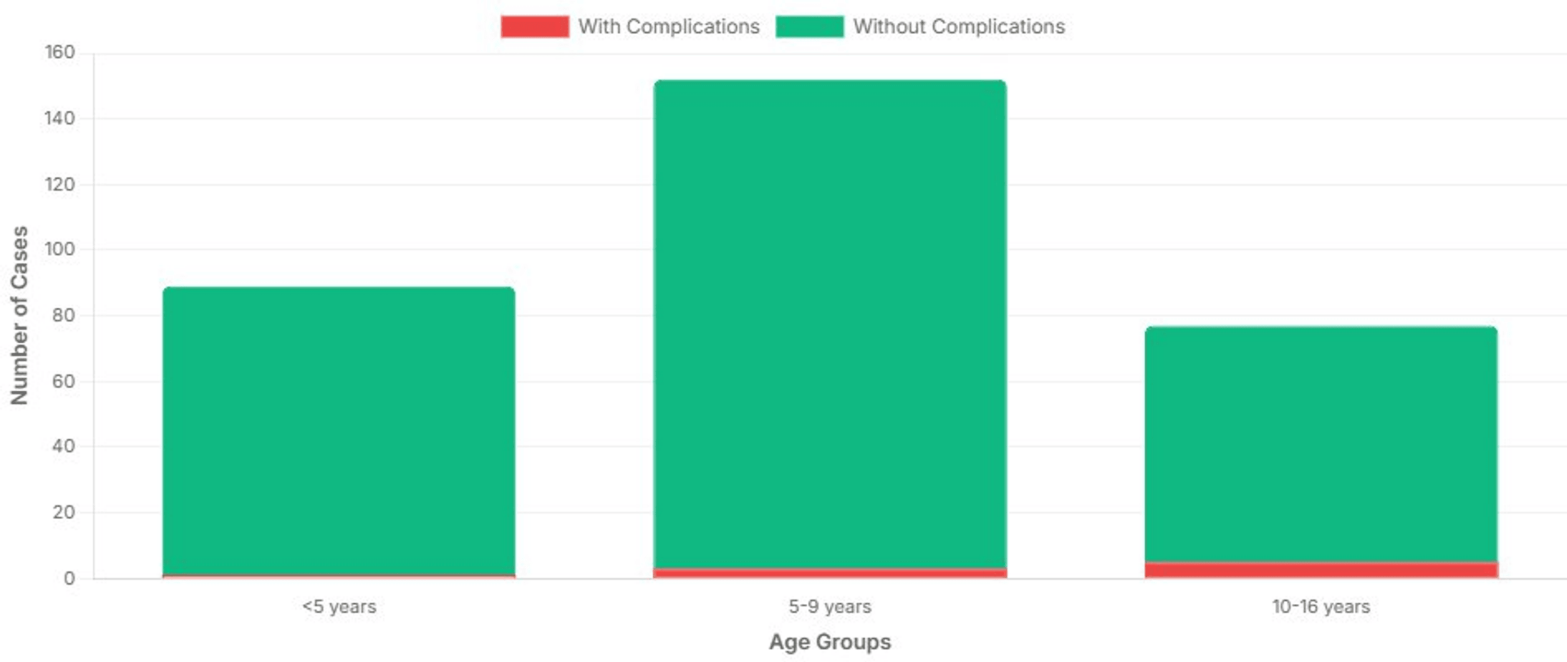
Comparison of complications by age group The x-axis shows age groups (<5 years, five to nine years, ≥10 years). The y-axis is labeled “Complications among IgM-confirmed cases (%)” and represents the percentage of children in each age band who developed at least one complication. Each bar (in a distinct colour for each age group) visually demonstrates the age gradient in complication risk.

The distribution of complications by age group is summarized in Figure 3, which shows the proportion (%) of IgM-confirmed mumps cases in each age band who developed at least one complication. The x-axis displays the three age groups (<5, five to nine years, ≥10 years), while the y-axis is labeled “Complications among IgM-confirmed cases (%)”. Each bar (displayed in a distinct colour for each age group) represents the percentage of confirmed cases in that age band with complications.

Risk by age bands

Complication risk increased markedly with age (Table 4, Figure 3). Among children <5 years, none of the 81 (0.0%; 95% CI 0.0-4.5%) developed complications. Among those aged five to nine years, four of 211 children (1.9%; 95% CI 0.5-4.8%) experienced complications. Among those aged ≥10 years, five of 26 children (19.2%; 95% CI 8.5-36.0%) had complications.

Compared with the five to nine year group, adolescents ≥10 years had substantially higher risk of complications (RR 10.1, 95% CI 3.0-34.3; Fisher’s exact p < 0.001). Formal risk estimates relative to the <5-year group could not be calculated due to 0 events, but the comparison was statistically significant (Fisher’s exact p = 0.001).

Complication proportions increased across age groups, with the highest observed proportion among children aged ≥10 years. However, estimates for this age group are based on a small sample size and are therefore imprecise, as reflected by wide confidence intervals.

**Figure 4:**
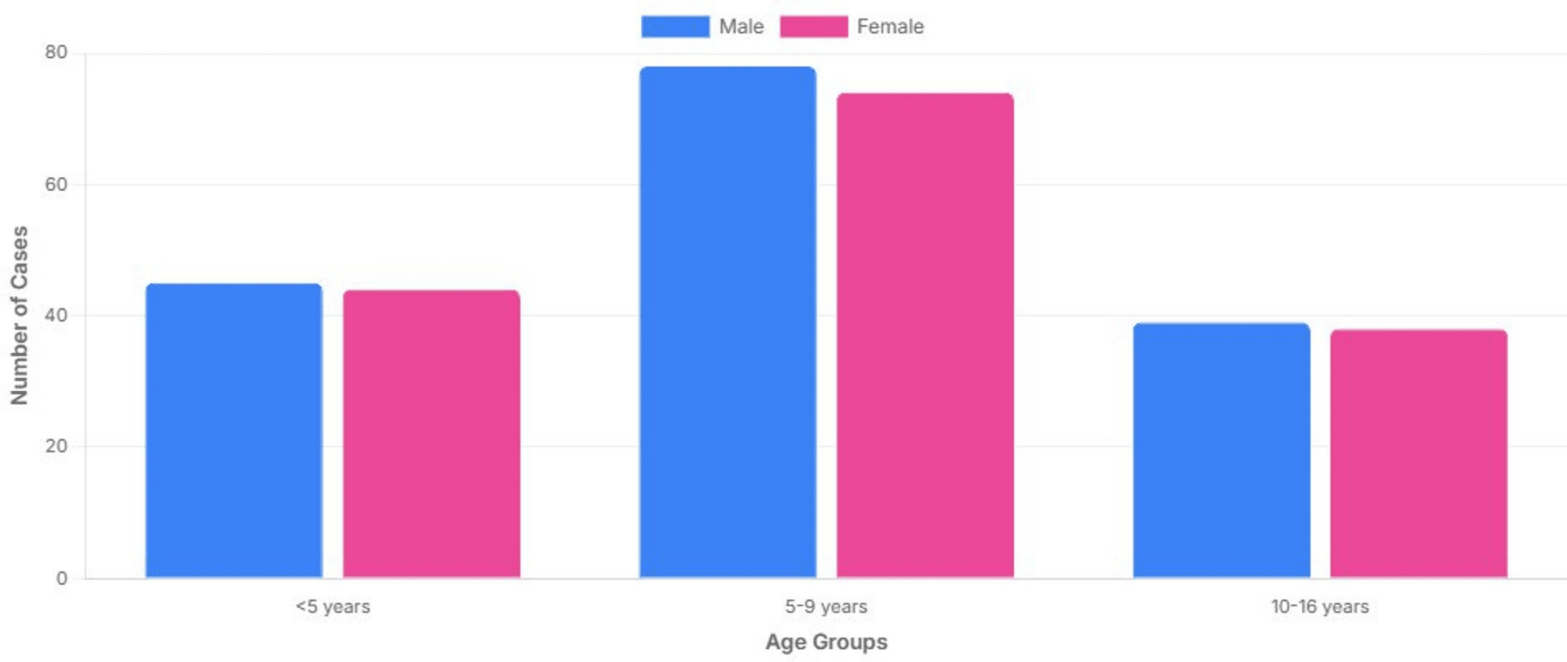
Age–sex pyramid of confirmed mumps cases in this study The x-axis shows the number of confirmed cases, with males on one side and females on the other; the y-axis is labeled “Age group (years)”. Bars indicate the distribution of cases across age bands by sex, illustrating the concentration of cases in the five-to-nine-year group.

These age-specific trends are visualized in Figure 4, an age-sex pyramid of confirmed cases. The x-axis shows the number of confirmed cases (separately for males and females on either side of the vertical axis), while the y-axis is labeled “Age group (years)”. The pyramid demonstrates the concentration of cases in the five-to-nine-year band with symmetric distribution among boys and girls.

Vaccination status

None of the 318 confirmed cases had documented receipt of a mumps-containing vaccine. Verification from immunization cards or registers was available for three of 318 children (0.9%), all of whom were unvaccinated for mumps. For the remaining 315 of 318 children (99.1%), parental recall consistently indicated absence of mumps-containing vaccination (Table 2).

In sensitivity analyses assuming up to 20% non-differential misclassification of vaccination status toward vaccinated, age-specific attack rate patterns and relative complication risks remained directionally unchanged and statistically robust.

## Discussion

In this district-level outbreak investigation, we found that school-aged children (five to nine years) bore the greatest burden of mumps, while complications clustered among adolescents ≥10 years. These findings align with global experience in settings where two-dose MMR coverage is suboptimal or waning immunity in older cohorts permits re-emergence of disease [2,4]. Our age-specific attack rates and complication profiles add quantitative detail to prior descriptive reports of mumps outbreaks in India [8,9].

The absence of complications among children <5 years in this outbreak is consistent with the hypothesis that maternally derived antibodies, although insufficient to prevent infection, may attenuate disease severity and reduce the risk of serious outcomes in early childhood [2]. In contrast, school-aged children and adolescents, who lack prior natural infection and have not received a mumps-containing vaccine under the national program, are more vulnerable to complications such as orchitis, meningitis, and pancreatitis [2,6]. Our data show a clear age gradient, with nearly one in five adolescents ≥10 years experiencing at least one complication. Complication proportions increased across age groups, with the highest observed proportion among children aged ≥10 years. However, estimates for this age group are based on a small sample size and are therefore imprecise, as reflected by wide confidence intervals. Because mumps is not a notifiable disease and surveillance is largely facility-based, milder and uncomplicated cases are less likely to be reported, leading to underestimation of true incidence. Conversely, hospital-based case ascertainment may preferentially capture more severe presentations, potentially overestimating the proportion of complications among reported cases.

Several factors likely contribute to the resurgence of mumps across India. First, MMR is not included in India’s UIP, and access to mumps-containing vaccines remains largely confined to the private sector. As a result, large cohorts of children remain fully susceptible. The lack of documented mumps vaccination among confirmed cases must be interpreted cautiously. Because vaccination history was predominantly based on parental recall, definitive classification of vaccination status is not possible. Second, mumps exhibits cyclical epidemic behavior; in the absence of routine vaccination, susceptible cohorts accumulate between epidemics, enabling periodic outbreaks [2,6]. Third, even where MMR is used, uptake of the second dose is often suboptimal, with many children receiving a single dose at nine to 12 months but missing the booster at 15 to 18 months [5]. Finally, the predominant Leningrad-Zagreb vaccine strain may not perfectly match circulating wild-type genotypes, potentially affecting vaccine effectiveness, although data from India are limited [4].

While mumps outbreaks have been documented in various parts of India over the past decade, reported outbreaks have generally involved smaller case counts or limited settings rather than large community-wide clusters. This investigation represents one of the larger systematically investigated mumps outbreaks reported recently in India, characterized by a substantial number of laboratory-confirmed cases and detailed attack rate estimation, which provides a more robust epidemiologic characterization. Furthermore, this outbreak highlights hospitalization patterns, demographic risk gradients, and complication profiles in a setting where the mumps vaccine is excluded from routine immunization, dimensions that have been underreported in prior Indian outbreak literature.

Our findings reinforce prior calls from Indian researchers and professional bodies for the introduction or strengthening of MMR vaccination in the public sector. The WHO position paper on mumps virus vaccines recommends routine inclusion of mumps-containing vaccines in national immunization schedules where mumps constitutes a public health problem [4]. Many countries have already implemented two-dose MMR programs with high coverage, leading to sustained reduction in mumps incidence and complications [2,4,10,11].

Economic considerations and programmatic context

Access to mumps-containing vaccines in India is heterogeneous and closely linked to socioeconomic status. Because MMR is not included in the Universal Immunization Programme, access is largely confined to the private healthcare sector, where vaccine uptake depends on household income, healthcare-seeking behavior, and urban proximity. Children from lower socioeconomic backgrounds, who primarily rely on public health services, are therefore less likely to receive MMR compared with children from higher socioeconomic groups accessing private pediatric care. Consequently, the absence of documented mumps vaccination among confirmed cases in this outbreak should be interpreted in the context of programmatic exclusion and differential access, rather than uniform vaccine refusal.

No formal cost-effectiveness analysis or budget-impact analysis was performed as part of this study. This study does not present a formal cost-effectiveness analysis; rather, it provides an illustrative, first-pass consideration of potential economic implications based on observed hospitalization burden. A frequent concern in policy discussions is the incremental cost of switching from MR to MMR in the UIP. However, the additional antigen cost, approximately ₹10-12 per dose in many Indian states, is modest when weighed against the costs of outbreak control, hospitalization, laboratory investigation, and caregiver productivity losses [5,12,14,15]. International cost-effectiveness analyses suggest that universal MMR programs are generally cost-saving or highly cost-effective, particularly when accounting for prevented complications and long-term sequelae [2,4,10].

In our setting, the median LOS of six days for complicated mumps implies substantial bed-day occupancy, opportunity costs, and caregiver time away from work. Even a conservative budget-impact model that considers only direct hospitalization costs during surge years suggests that adding mumps to the existing MR platform could be fiscally neutral or cost-saving over a multiyear horizon, especially if school-based catch-up campaigns in the five-to-15-year age group are implemented alongside routine infant immunization [4,10].

Public health and policy implications

The findings add district-level, outbreak-based evidence to the limited Indian literature on mumps burden and may inform future surveillance priorities and policy deliberations when considered alongside broader national data. Our data underscore several actionable priorities. The findings support the plausibility of benefit from mumps-containing vaccination but are insufficient on their own to justify national policy change. First, integrating MMR into the UIP with a two-dose schedule at nine to 12 and 15 to 18 months, aligned with IAP recommendations, would close the current programmatic gap depicted in Table 5 [11,12,14]. Second, targeted catch-up vaccination for children aged five to 15 years in outbreak-prone districts, delivered through schools and outreach sessions, could rapidly reduce the pool of susceptible individuals and prevent recurrent epidemics [4,12,15,16]. Third, strengthening surveillance for mumps by making it a notifiable disease and incorporating routine laboratory confirmation and occasional genotyping would enable more accurate burden estimates and earlier detection of clusters [4,7-10]. 

**Table 5:** Comparison of UIP vs. IAP immunization schedule, and the EPI chart ✔ = vaccine included in the respective schedule; Ø = vaccine not included in the respective schedule. UIP: Universal Immunization Programme (Government of India); IAP: Indian Academy of Pediatrics (schedule); EPI: Expanded Programme on Immunization; BCG: Bacillus Calmette–Guerin vaccine; OPV: oral polio vaccine; IPV: inactivated polio vaccine; DPT: diphtheria–pertussis–tetanus vaccine; Hib: *Haemophilus influenzae* type B vaccine; PCV: pneumococcal conjugate vaccine; MMR: measles–mumps–rubella vaccine; Varicella: varicella (chickenpox) vaccine; Hep B: hepatitis B vaccine; Hep A: hepatitis A vaccine; Typhoid: typhoid vaccine.

Vaccine	UIP	IAP
BCG	✔	✔
OPV/IPV	✔	✔
Hep B	✔	✔
DPT	✔	✔
Hib	✔	✔
Pentavalent	✔	✔
Rotavirus	✔	✔
PCV	✔	✔
Measles	✔	✔
Rubella	✔	✔
Mumps	Ø	✔
MMR	Ø	✔
Varicella	Ø	✔
Hep A	Ø	✔
Typhoid	Ø	✔

**Figure 5:**
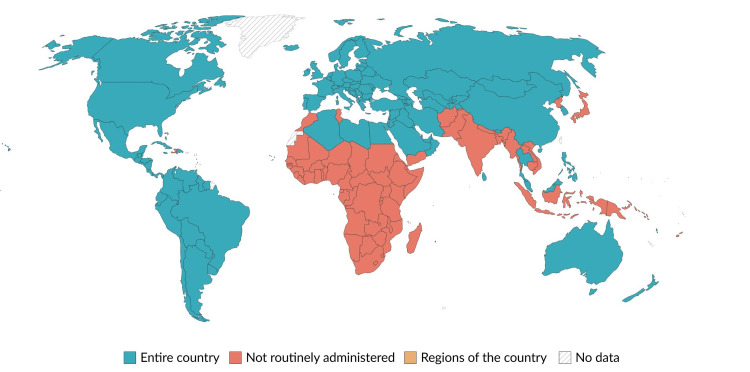
World map highlighting the inclusion of MMR in national immunization programs globally, with India as the exception This is an original illustration created by the authors. World map highlighting the inclusion of MMR in national immunization programs globally, with India as the exception. Map template based on Natural Earth public domain data. Countries are shaded according to MMR inclusion status in national immunization programs. Data sources: WHO/UNICEF immunization schedule data [10,11]. Map created by authors using Natural Earth vector data [17]. MMR: measles–mumps–rubella; WHO: World Health Organization: UNICEF: United Nations Children's Fund

India remains one of the few countries globally that has not yet incorporated mumps-containing vaccines into its national immunization program (Figure 5), despite widespread international adoption of MMR vaccination schedules.

Epidemiologic acknowledgment

We acknowledge that the estimation of attack rates in this study does not meet the strict epidemiologic requirement that numerators and denominators arise from the same fully enumerated population. Laboratory-confirmed cases were ascertained through facility-based surveillance, whereas denominators were derived from district population projections. As such, the calculated rates should not be interpreted as true population incidence but rather as conservative, facility-based indicators of relative age-specific burden during the outbreak period.

Limitations

This study has several limitations. First, being facility-based, it likely underestimates the true burden of mumps, as milder or asymptomatic community cases that did not seek care were not captured [2,6]. Selective testing of hospitalized or more severe presentations may have introduced severity bias, potentially inflating observed complication proportions and median length of hospital stay among laboratory-confirmed cases. Consequently, complication rates and LOS reported in this study should be interpreted as reflecting the burden among confirmed, healthcare-seeking cases rather than the full spectrum of community infection. Small sample sizes in older age strata limited the precision of age-specific complication estimates. Second, laboratory confirmation relied on IgM ELISA rather than reverse transcription polymerase chain reaction (RT-PCR) or paired sera, which may miss very early infections or yield false-negative results in certain windows [2,4,6]. IgM sensitivity varies with the timing of specimen collection and may be reduced in early infection, reinfection, or secondary immune responses, potentially resulting in false-negative results. Consequently, some true mumps cases may have been misclassified as unconfirmed and excluded from the analytic cohort. This limitation is likely to bias incidence estimates downward and further supports the interpretation of our findings as conservative, facility-based estimates rather than complete outbreak enumeration. Third, vaccination history was partly based on parental recall, introducing potential misclassification; furthermore, claims regarding complete susceptibility or definitive absence of prior vaccination cannot be made with confidence. However, sensitivity analyses suggest that even moderate misclassification does not alter the key age-specific findings. Fourth, the investigation was restricted to a single district; while age patterns were consistent with international literature, caution is warranted in extrapolating attack rates and complication proportions to other regions [2,4,10,14,15].

This study did not collect individual-level socio-economic indicators; therefore, socio-economic gradients in vaccine access could not be formally analyzed, and inferences are based on established health-system patterns rather than direct measurement. The use of district-level population denominators with facility-based numerators represents a methodological limitation. This approach does not account for healthcare-seeking behavior, hospital catchment boundaries, or under-ascertainment of mild and community-managed cases, and therefore likely underestimates true incidence while preserving relative age-pattern comparisons. The absence of a formal economic or budget-impact analysis limits conclusions regarding the economic consequences of vaccination policy changes. The single-district, facility-based design limits the ability to generalize findings or directly extrapolate to national immunization policy decisions.

Future work should prospectively link facility and community surveillance, incorporate RT-PCR and genotyping, and evaluate school-based MMR catch-up campaigns with coverage and outcome monitoring.

## Conclusions

This outbreak demonstrates a substantial, age-resolved burden of mumps among school-aged children in an Indian district where the mumps vaccine is not part of the UIP. Complications were concentrated in adolescents ≥10 years; all confirmed cases lacked documented mumps vaccination. These findings may help inform future formal economic evaluations, but do not constitute evidence of cost neutrality or cost savings. These findings add district-level, outbreak-based evidence to the limited Indian literature on mumps burden and may inform future surveillance priorities and policy deliberations when considered alongside broader national data.

The continued exclusion of mumps from the national immunization program represents a critical shortcoming in preventive child health. Introducing a two-dose MMR schedule into India’s UIP, coupled with school-age catch-up vaccination and strengthened surveillance, is a data-supported intervention likely to reduce mumps complications and hospitalizations in similar settings. The findings provide supportive epidemiologic evidence that may inform ongoing national deliberations regarding inclusion of mumps-containing vaccines in the UIP. Policy decisions should integrate these findings with national surveillance data, feasibility assessments, and formal economic evaluations.
